# Porosity/Cement Index and Machine Learning Models for Predicting Tensile and Compressive Strength of Cemented Silt in Varying Compaction Conditions

**DOI:** 10.3390/ma19030498

**Published:** 2026-01-27

**Authors:** Jair Arrieta Baldovino, Oscar E. Coronado-Hernández, Yamid E. Nuñez de la Rosa

**Affiliations:** 1 Department of Civil Engineering, Universidad de Cartagena, Cartagena de Indias 130015, Colombia; 2 Instituto de Hidráulica y Saneamiento Ambiental, Universidad de Cartagena, Cartagena de Indias 130001, Colombia; ocoronadoh@unicartagena.edu.co; 3 Faculty of Engineering and Basic Sciences, Fundación Universitaria Los Libertadores, Bogotá 110231, Colombia

**Keywords:** soil-cement, machine learning, porosity-cement index, ground improvement, optimization

## Abstract

This study investigates the mechanical response of cemented silt subjected to 28 days of curing by integrating two predictive methodologies: porosity–cement index (η/C_iv_) and machine learning (ML) models. The soil was compacted over a wide range of molding water contents and dry densities, including optimum and off-optimum states, and stabilized with varying cement contents. Unconfined compressive strength ($q_{u}$) and splitting tensile strength ($q_{t}$) were evaluated as functions of cement dosage, curing time, porosity, water content, and the specific gravities of the soil and cement. The η/C_iv_ index demonstrated a strong predictive capability for both $q_{u}$ and $q_{t}$, with determination coefficients exceeding 0.980, and exhibited the expected power-law decay with increasing η/C_iv_. ML algorithms—particularly Gaussian Process Regression with a Matern 5/2 kernel—outperformed the empirical model, achieving *R*^2^ values of 0.963 (validation) and 0.997 (testing) for $q_{u}$ prediction. The $q_{t}$ model similarly reached *R*^2^ = 0.984–0.988, demonstrating high generalization and stability across curing and compaction conditions. Experimental results revealed substantial strength gains with decreasing η/C_iv_, with $q_{u}$ increasing from 100 kPa at η/C_iv_ = 46 to 2900 kPa at η/C_iv_ = 19, while $q_{t}$ rose from 10–15 kPa to 300 kPa across the same range.

## 1. Introduction

Since 2007, Consoli et al. [1] have used different approach to estimate the strength, durability, and stiffness of various stabilized soils, including those treated with lime and cement, as well as those incorporating different waste materials and alternative binders. The porosity-cement ratio was introduced initially by Larnach [2] in 2019, but it was later named the void/cement ratio when considering this index to estimate the unconfined compressive strength of cemented sand. The porosity–cement ratio exhibits a close correlation with both the intrinsic properties of the soil and the characteristics of the binders employed [1]. This approach has been applied to evaluate the geotechnical properties of clean sands, silts, and clays. The empirical estimation of unconfined compressive strength ($q_{u}$) and ($q_{t}$) is often expressed in the following form (Equation (1)):

(1) $$q_{u}\;or\;q_{t}=A{\left[\frac{\eta }{{\left(C_{iv}\right)}^{x}}\right]}^{-B}$$

*A*, *x*, and *B* values depend on the properties of the soil and cement, as well as their interaction. Constant *A* is expressed in kPa. The index η/C_iv_ expresses, in a single parameter, the combined influence of both porosity and binder content on the mechanical strength of a material [3,4]. Thus, the relative importance of each factor can be adjusted through the exponent x; that is, when the effect of porosity is more significant, x assumes a value lower than 1.0. For several studies, the coefficient of determination (*R*^2^) values obtained for estimating $q_{u}$, $q_{t}$, stiffness ($G_{o}$), and accumulated loss of mass (ALM) as functions of the η/C_iv_ ratio are generally high (e.g., [4,5,6]).

Recent research has demonstrated that porosity–binder indices serve as a powerful and generalizable framework for predicting strength, stiffness, and durability across a wide range of stabilized soils and geomaterials. Nierwinski et al. [7] and Baldovino et al. [8] further extended the applicability of this approach by demonstrating that the porosity/binder index yields highly predictive models for $q_{u}$ and ultrasonic stiffness in clay treated with mineral binders, industrial waste additives, and biopolymers, although gel-based systems such as xanthan gum required significantly lower exponents due to their distinct microstructural bonding mechanisms. Alibeigibeni et al. [9] emphasize that recycled concrete aggregates (RCAs) represent a sustainable alternative to natural aggregates, although their higher porosity and adhered mortar typically reduce mechanical strength and durability. Nonetheless, pretreatment strategies, optimized mix designs, and the incorporation of supplementary cementitious materials substantially improve RCA performance. In addition, recent studies have highlighted the increasing applicability of machine learning (ML) techniques for predicting the mechanical performance of stabilized soils, particularly when sustainable and waste-based binders are incorporated. Sridhar et al. [10] developed a robust ML framework to predict the unconfined compressive strength and California Bearing Ratio (CBR) of lateritic soils stabilized with industrial wastes such as red mud, copper slag, and iron ore tailings, demonstrating that Random Forest and multilayer perceptron models achieved high predictive accuracy (*R*^2^ > 0.90), with SHAP analysis identifying curing time, maximum dry density, and waste dosage as dominant factors. Similarly, Daimary et al. [11] employed artificial neural network (ANN) models to estimate $q_{u}$, maximum dry density, and optimum moisture content of lateritic soils treated with agro-industrial wastes (cement kiln dust and rice husk ash), achieving *R*^2^ values up to 0.98 and confirming the capability of ANN models to capture complex nonlinear soil–binder interactions. Extending ML applications to solid waste–cement systems, Tu et al. [12] proposed an optimized hybrid extreme gradient boosting model for CBR prediction, which outperformed conventional ML approaches and identified cement content, fine-grained fraction, and calcium oxide content as the most influential variables.

Similarly, Hanafi et al. [13] confirmed that an adjusted porosity index (exponent 0.32) unifies the prediction of $q_{u}$ initial shear modulus ($G_{o}$), and ALM in alluvial clay blended with cement and marble dust, underscoring the index’s capacity to accommodate sustainable binder replacements. Muñoz et al. [14] also demonstrated that both η/C_iv_ accurately correlate $q_{u}$, $q_{t}$, and ALM in soil–cement–glass polishing waste mixtures, reducing reliance on labor-intensive trial-and-error procedures and supporting the development of optimized geomaterials. In contrast, Ribeiro et al. [15] revealed that although η/C_iv_ effectively captures early-age strength in dredged sediments treated with lime and cement, long-term degradation due to ettringite formation can override mechanically favorable porosity–binder states, emphasizing chemical durability as a critical complement to index-based design. Finally, Haider et al. [16] showed that mixtures incorporating PET shreds and reduced cement content still follow a porosity–binder trend for CBR (California Bearing Ratio), ALM, and shear modulus, demonstrating that the index remains valid even when the binder phase is mechanically augmented with polymeric reinforcement.

In parallel with the widespread use of porosity–binder indices, there has been a rapid expansion of machine learning (ML) methods for predicting the strength and related properties of stabilized soils, often with high accuracy and increasing levels of interpretability. Focusing on nano-silica-stabilized fine-grained soils, Thapa et al. [17] proposed deep learning models (CNN, LSTM, and RNN) to predict $q_{u}$ from an extensive database of CI, MI, and CL–ML soils, showing that a CNN-based model, embedded in a graphical user interface, can reliably capture strength gains of several hundred percent associated with nano-silica dosage and curing time. Kumar et al. [18] compared Gradient Boosting Machine, Random Decision Forest, non-parametric regression, and decision trees for nano-doped fly-ash-treated clayey soils and identified GBM as the most accurate and interpretable model, using sensitivity, monotonicity, and SHAP analyses to highlight the dominant roles of curing days, maximum dry density, and sodium hexametaphosphate content in $q_{u}$ development.

Moving beyond purely data-driven approaches, Yang et al. [19] introduced physics-informed ML frameworks for cement-stabilized soils—residual-learning hybrid corrector models and physics-informed neural networks—embedding a modified water–cement ratio model into training; the best-performing PINN-based multi-scale DNN achieved high *R*^2^ and, through SHAP, quantified the relative importance of cement content, water content, and clay fraction. A second contribution by Thapa et al. [20] addressed na–silica-stabilized infinite slopes, utilizing an Optuna-optimized hybrid RNN–CNN–LSTM classifier to achieve 99% accuracy. The study also employed XAI–SHAP techniques to demonstrate that cohesion, nano-silica content, and slope angle are the primary factors governing slope stability. At the material-design scale, Yao et al. [21] developed a zebra-optimized XGBoost model to predict compressive strength in cohesive soils stabilized with industrial by-product geopolymers, demonstrating that calcium oxide, silicon dioxide, and curing age dominate strength evolution, enabling practical mix-design windows based on chemical composition.

Similarly, Luo et al. [22] built a data-driven framework for $q_{u}$ prediction in stabilized soils and identified a deep forest model as the most accurate; SHAP analysis and response-surface interpretations revealed that NaOH and GGBS contents are key drivers of strength in alkali-activated systems. Wang [23] adopted an automated ML strategy based on decision trees enhanced with metaheuristic optimizers (DTSH, DTTS, DT), showing that the DTSH hybrid can achieve extremely high *R*^2^ values across diverse stabilized-soil datasets. Hu et al. [24] combined gradient boosting decision trees with a genetic algorithm to predict and simultaneously optimize the $q_{u}$ of GGBS–fly-ash geopolymer-stabilized soils, demonstrating the predominance of binder content, curing age, and GGBS/FA ratio in strength development. Teodoru et al. [25] integrated laboratory testing with Bayesian-optimized ML models for cement-treated clayey silt, identifying Random Forest as the optimal predictor and coupling it with uncertainty quantification and SHAP-based interpretation to produce a web-based $q_{u}$ prediction tool. Along similar lines, Linganagoudar et al. [26] used decision trees, Random Forest, and multilayer perceptrons to predict $q_{u}$ in lateritic soils treated with cement and FGD gypsum, showing that the nonlinear interactions between cement–gypsum dosage and curing duration can be captured with *R*^2^ ≈ 0.98, with curing time emerging as the most influential factor. Finally, Mustafa et al. [27] explored SVR and decision tree models for both stabilized and unstabilized soils, demonstrating that grain-size distribution and moisture content dominate $q_{u}$ in natural soils. In contrast, the type and content of stabilizers, along with density and moisture state, become the primary predictors in stabilized mixtures.

Given the growing evidence that both porosity–binder indices and machine learning frameworks provide powerful tools for interpreting and predicting the behavior of stabilized soils, this study aims to integrate these two approaches to evaluate the mechanical response of cemented silt compacted at different moisture and density conditions. Specifically, the objective is to assess the extent to which the porosity–cement index and a set of supervised machine learning models can accurately estimate the unconfined compressive strength ($q_{u}$) and splitting tensile strength ($q_{t}$) of mixtures cured for 28 days. To achieve this, the analysis incorporates key mixture-design variables—including cement content, curing time, molding density, water content, specific gravities, and porosity—to identify the relative predictive capacity of each method and determine whether data-driven learning can complement or outperform the classical porosity–cement framework. Although recent studies have combined porosity–binder indices with machine learning techniques for predicting the behavior of stabilized soils, the present research introduces a distinct contribution by explicitly integrating the porosity–cement index (η/C_iv_) as a physically based parameter within a comprehensive ML framework. Unlike purely data-driven approaches, this study evaluates both optimum and non-optimum compaction conditions and directly compares classical η/C_iv_ formulations with advanced ML models.

## 2. Experimental Program

The experimental plan is primarily based on comparing the application of the porosity-to-cement ratio (η/C_iv_) and machine learning methods to estimate the unconfined compressive strength ($q_{u}$) and the splitting tensile strength ($q_{t}$) compacted in strategic molding points variating the water content and dry unit weight, also compatible with optimum compaction conditions of optimum water content (OWC) and maximum dry unit weights (MDD) of silty soil improved with Portland cement. The data analysis of the porosity-to-cement index in the optimal compaction conditions was reported by Baldovino et al. [28], and the report of the influence of the porosity/cement index for estimating $q_{u}$ and $q_{t}$ in non-optimal compaction conditions was published by Baldovino et al. [29].

A total of n = 408 tests were performed for the determination of $q_{u}$ and $q_{t}$ at non-optimal conditions (i.e., fixing dry unit weight and water content) and n = 216 tests for estimating $q_{u}$ and $q_{t}$ at optimal compaction conditions (i.e., OMC and MDD).

Although this study focuses on a single silty soil stabilized with Portland cement, the porosity–cement index adopted herein is a generalized framework previously validated for different soil types. Nevertheless, the specific empirical parameters obtained in this research are material-dependent and require recalibration when applied to other soils or binders.

### 2.1. Materials

The materials used in this study included silty soil and high-early-strength Portland cement, along with distilled water used in the preparation of test specimens.

**Figure 1:**
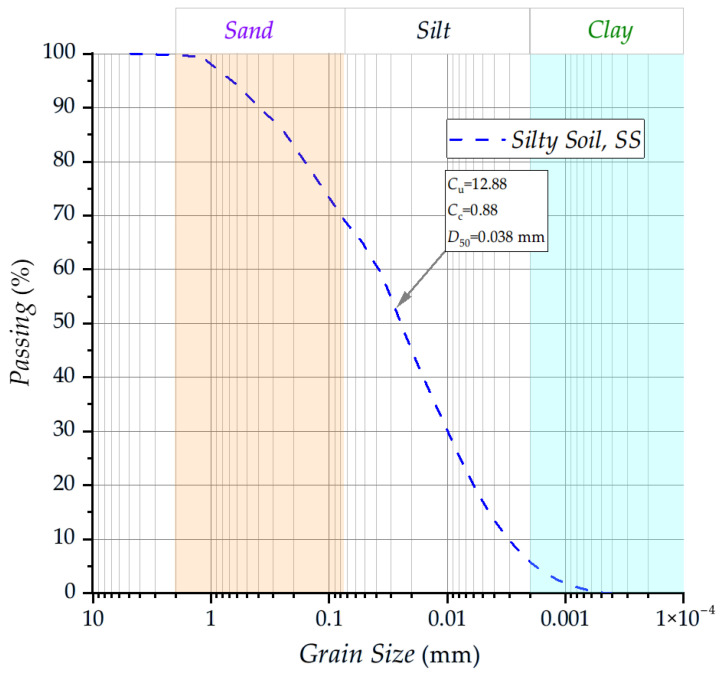
The granulometric curve of the silty soil sample.

In concordance with Baldovino et al. [28,29], the soil used in this study corresponds to a high-plastic silt (Unified Soil Classification System: MH), characterized by a liquid limit of 50.82% and a plastic limit of 35.96%, resulting in a plasticity index of 14.86% (ASTM D4318 [30]). Its specific gravity is 2.62 (ASTM D854 [31]). The particle-size distribution (Figure 1) indicates a predominantly fine-grained material, composed of 60% silt-sized particles and only 5% clay, while the sand fraction (5% coarse, 12% medium, and 18% fine) represents a minor portion of the matrix (NBR 6502 [32]). The effective size (D_10_) of 0.003 mm and mean particle diameter (D_50_) of 0.038 mm, together with a uniformity coefficient (C_u_ = 12.88) and a coefficient of curvature (C_c_ = 0.88), reflect a well-graded non-plastic silty structure with limited coarse particles. In its natural state, the soil exhibits an unconfined compressive strength ($q_{u}$) of 104.58 kPa and a splitting tensile strength ($q_{t}$) of 16.62 kPa, yielding a $q_{t}$/$q_{u}$ ratio of 0.16, which is typical of weakly bonded fine-grained soils. Direct shear testing (ASTM D3080 [33]) yields a friction angle of 26° and a cohesion value of 23 kPa, parameters consistent with those of a moderately cohesive silt. Visually, the soil presents a characteristic yellow coloration.

The Portland cement used in this study exhibits a chemical composition typical of high-calcium binders, characterized by a predominance of CaO (60.76%), followed by significant contents of SiO_2_ (18.96%) and Al_2_O_3_ (4.30%), which are essential for the formation of calcium silicate hydrates (C–S–H) and calcium aluminate phases responsible for strength development. Minor oxides, including Fe_2_O_3_ (2.95%), MgO (3.26%), and SO_3_ (3.18%), fall within expected ranges for commercial cements, ensuring adequate sulfate balance and volumetric stability. The cement contains a low insoluble residue (0.77%), indicative of high clinker purity. Compressive strengths of 44.7 MPa at 7 days and 54.2 MPa at 28 days demonstrate mechanical performance. The fineness of 0.04% contributes to increased reactivity by providing a larger surface area for hydration reactions. Additionally, the specific gravity of 3.11 (NBR 16605 [34]) aligns with values typical of Portland cements. The Supplementary Materials contain the information used in this study.

### 2.2. Specimen Molding and Preparation

All specimens, both for unconfined compressive and splitting tensile tests, were compacted by static pressing in three layers within steel cylindrical molds. The specimens prepared for compression and tensile strength tests had a height of 100 mm and a diameter of 50 mm. After compaction, all specimens were sealed and cured in a humid chamber for 7, 14, or 28 days (depending on the compaction conditions) prior to conducting the compression and tensile tests. The details of the curing conditions and mixture compositions are summarized in Table 1 and Table 2.

**Figure 2:**
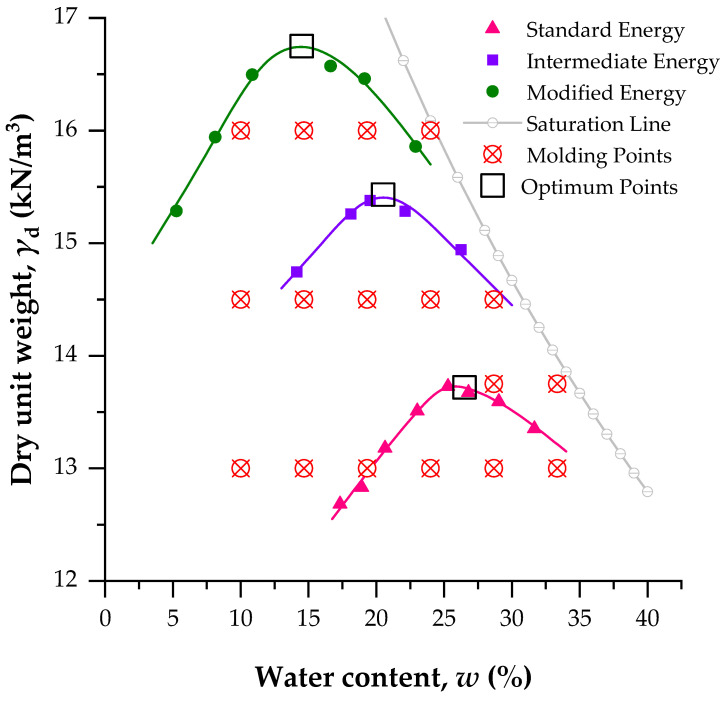
The compaction curve of soil (at standard, intermediate, and modified energies), saturation line of 100%, molding points, and optimum points.

Figure 2 shows the compaction curves of the soil under standard, intermediate, and modified energies, along with the 100% saturation lines and the molding points of the soil–cement specimens. The molding points are divided into two groups: points prepared at a constant water content with varying dry unit weights, and points corresponding to optimum compaction conditions (i.e., MDD and OWC).

### 2.3. Unconfined Compressive and Splitting Tensile Protocols

The unconfined compression tests were conducted in accordance with ASTM D2166/D2166M-16 [35], while the indirect tensile (Brazilian) tests followed the procedures outlined in ABNT NBR 7222 [36]. All mechanical tests ($q_{u}$ and $q_{t}$) were carried out using a Geotechnik testing machine with a loading rate of 1.14 mm/min and a maximum capacity of 20 kN. Data acquisition was performed with a force sensitivity of 0.25 N, ensuring precise measurement of load–deformation behavior throughout the tests.

## 3. Machine Learning Methodology

In this study, each category of machine learning model was employed to predict the values of $q_{u}$ and $q_{t}$. Table 3 summarizes the selected algorithm types. With respect to interpretability, linear models, decision trees, and Support Vector Machines (SVM), efficiently trained linear models are relatively easy to interpret, meaning that the influence of the predictors can be assessed rapidly and predictions can be generated efficiently [37]. In contrast, SVM models with nonlinear kernels offer limited interpretability. The remaining machine learning models—Gaussian Process Regression, kernel-based models, ensembles of trees, and neural networks—are generally more challenging to interpret [37]. Nevertheless, both Gaussian Process Regression and neural networks provide strong predictive performance for $q_{u}$ and $q_{t}$. Table 1 and Table 2 present the information regarding the dataset employed for implementing the ML models.

Table 4 presents the formulas associated with the machine learning algorithms employed in this study. These formulas are organized into eight categories; however, depending on the model type, several algorithmic variants may be derived based on their internal parameters. In total, twenty-eight ML models were evaluated. The hyperparameter optimization framework provided in MATLAB R2024b was adopted to obtain the best statistical performance for all models. A summary of the underlying formulas is provided, taking into account their relative complexity. Accordingly, several clarifying observations are included to facilitate the interpretation of the machine learning results.

As a crucial aspect of the methodology, it is essential to select appropriate predictors together with their corresponding responses when applying machine learning models. Table 5 presents the predictor–response pairs used in this study. In total, five predictors were identified, as shown in the experimental program of this research (see Section 2). These predictors are utilized for computing the two responses of the ML algorithms ($q_{u}$ and $q_{t}$).

During the development of the ML models, a 5-fold cross-validation scheme was employed (80% of the dataset), with 20% of the dataset reserved for testing purposes to minimize the risk of overfitting.

## 4. Results and Discussions

### 4.1. Effects of Porosity-to-Cement Index on Unconfined Compressive and Splitting Tensile Strength Considering the Optimum Compaction Conditions

**Figure 3:**
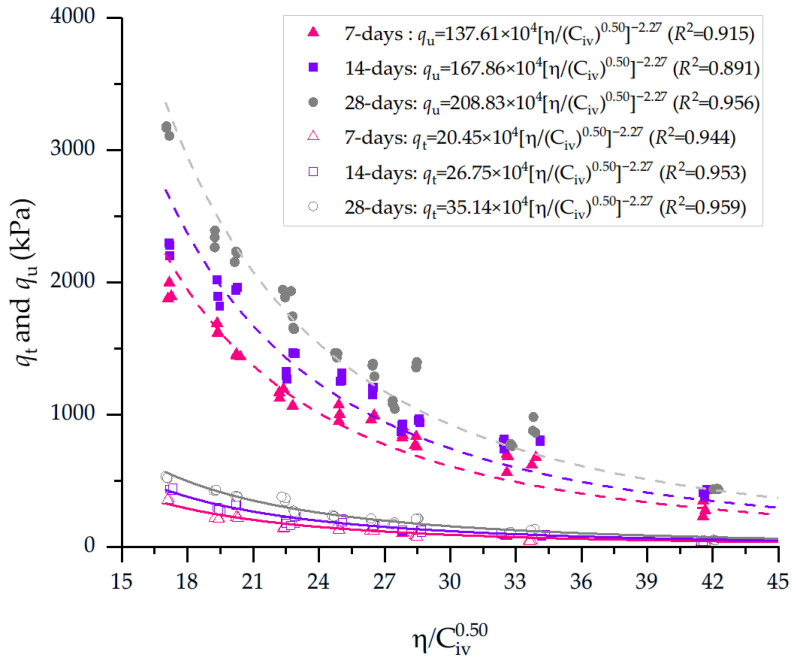
Results of unconfined compressive strength and splitting tensile strength of soil–cement blends considering the porosity-to-cement ratio index (adjusted to 0.50) and 7, 24, and 28 days of curing. Blends were compacted at MDD and OMC (Table 2).

Figure 3 correlates the porosity/cement index and $q_{u}$ and $q_{t}$ for 7, 14, and 28 days, when the soil was compacted at MDD and OWC, as presented in detail in Table 6. The experimental results of $q_{u}$ confirm a strong inverse relationship between the porosity–cement index (η/C_iv_) and $q_{u}$ of the cemented silt, consistent with the classical porosity–cement framework. For specimens cured for 28 days under standard compaction energy, η/C_iv_ decreases from approximately 37 for mixtures with 3% cement to about 22, 16, and 13 for cement contents of 5%, 7%, and 9%, respectively. In parallel, $q_{u}$ increases from roughly 435 kPa at 3% cement to around 775 kPa at 5%, 1080 kPa at 7%, and 1450 kPa at 9%. A similar trend is observed for the intermediate and modified compaction energies: for 28-day curing and 5% cement, η/C_iv_ decreases from approximately 22.6 (standard) to 17.1 (intermediate) and 14.0 (modified), while $q_{u}$ increases from 770 kPa to 1780 kPa and then to 1900 kPa. These results indicate that both higher cement content and higher compaction energy primarily act by reducing η/C_iv_, densifying the soil skeleton, and increasing the volumetric fraction of cement, which collectively enhance q_u_.

As expected, lower values of η/C_iv_—resulting from reduced porosity and/or higher volumetric cement contents—produce consistently higher $q_{t}$ values. For specimens compacted under Standard energy at 28 days, η/C_iv_ decreased from approximately 37.1 to 12.8, yielding a corresponding increase in $q_{t}$ from 50 kPa (3% cement) to 236 kPa (9% cement). Under Intermediate energy, the same curing age showed an even more pronounced response: η/C_iv_ ranged from 28.0 to 9.9, while $q_{t}$ increased from 124 kPa to 386 kPa, demonstrating the dual effect of higher density and improved cement bonding. The trend was strongest for the Modified compaction energy, in which η/C_iv_ dropped to values as low as 9, resulting in $q_{t}$ levels that reached 525 kPa for 9% cement at 28 days. A similar pattern was observed for shorter curing times. At 14 days, Standard-energy specimens exhibited $q_{t}$ values ranging from 38 to 213 kPa as η/C_iv_ decreased from 36.5 to 13.1, while Intermediate and Modified energies again produced enhanced strength, with $q_{t}$ reaching 327 kPa and 436 kPa, respectively, for η/C_iv_ values near 8–10. After only 7 days of curing, the relationship remained consistent: $q_{t}$ increased from 36 kPa to 133–170 kPa under Standard energy (η/C_iv_ from 36.5 to 13.0), and up to 365 kPa under Modified energy (η/C_iv_ = 8.1–10.3).

Table 6 presents the equations governing the unconfined compressive and splitting tensile strengths of compacted soil–cement blends.

### 4.2. Effects of Porosity-to-Cement Index on Unconfined Compressive and Splitting Tensile Strength Considering the Non-Optimum Compaction Conditions

**Figure 4:**
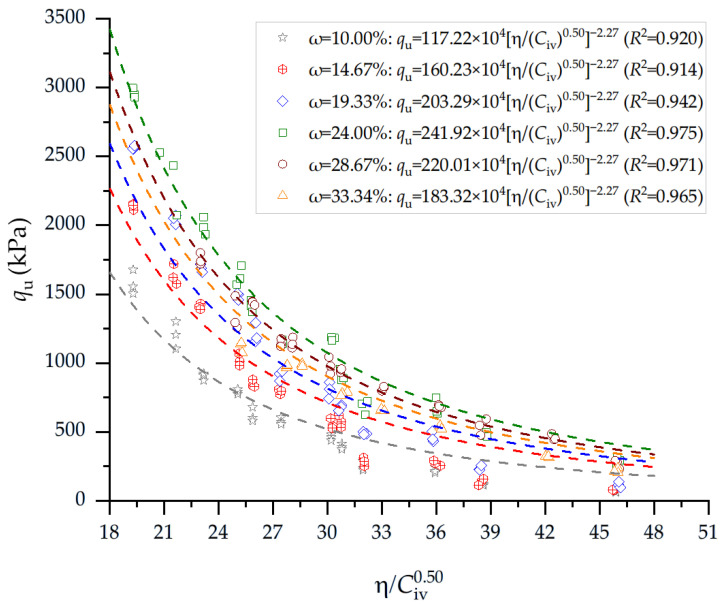
Results of unconfined compressive strength of soil–cement blends considering the porosity-to-cement index (adjusted to 0.50) and 28 days of curing. Blends were compacted at molding dry unit weight and variable water content (Table 1).

Figure 4 presents the results of the $q_{u}$ of soil–cement blends considering the porosity-to-cement index (adjusted to 0.50) and 28 days of curing for the blends compacted at the molding dry unit weight and with variable water content, as presented in Table 1. The results reveal a clear and systematic influence of the $\eta /C_{iv}^{0.50}$ index on the 28-day $q_{u}$ of the cemented silt, demonstrating that reductions in $\eta /C_{iv}^{0.50}$ are consistently associated with substantial strength gains across all moisture conditions. For specimens compacted near 10% water content, $q_{u}$ values increased from approximately 60–72 kPa at $\eta /C_{iv}^{0.50}\;$= 45.9 to more than 600 kPa when $\eta /C_{iv}^{0.50}$ decreased to 27.5 and further exceeded 900 kPa when $\eta /C_{iv}^{0.50}$ reached 23.2. At slightly higher moisture levels (14%), the same trend persisted: $q_{u}$ increased from 75–82 kPa at $\eta /C_{iv}^{0.50}$ = 45.7 to about 550–620 kPa when $\eta /C_{iv}^{0.50}$ decreased to 30.7 and then rose sharply to 770–810 kPa at $\eta /C_{iv}^{0.50}$ = 27.4; further reductions to ~23.0 resulted in strengths between 1.39 and 1.43 MPa. At moisture contents around 19%, high strengths were also obtained, with $q_{u}$ rising from 115 kPa at $\eta /C_{iv}^{0.50}$ = 46.1 to 475 kPa at $\eta /C_{iv}^{0.50}$ = 35.8 and reaching 925 kPa when $\eta /C_{iv}^{0.50}$ approached 27.4. Subsequently, strengths greater than 2.0 MPa were recorded at *n* $\eta /C_{iv}^{0.50}$ = 19.3. This behavior was consistent for higher molding moisture (24–29%), where initial $q_{u}$ values remained below 300 kPa at $\eta /C_{iv}^{0.50}$ = 46.0 but increased to 800 kPa when $\eta /C_{iv}^{0.50}$ fell to ~36.0 and exceeded 1.1–1.4 MPa at $\eta /C_{iv}^{0.50}$ = 25.8; ultimately, values between 2.0 and 3.0 MPa were achieved at $\eta /C_{iv}^{0.50}$ = 19.3.

**Figure 5:**
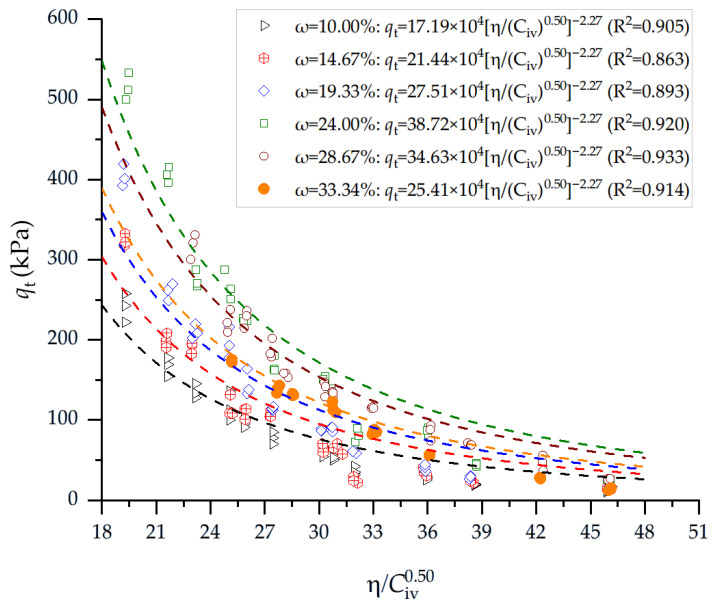
Results of splitting tensile strength of soil–cement blends considering the porosity-to-cement index (adjusted to 0.50) and 28 days of curing. Blends were compacted at molding dry unit weight and variable water content (Table 1).

Figure 5 presents the results of $q_{t}$ of soil–cement blends considering the $\eta /C_{iv}^{0.50}$ index and 28 days of curing for the blends compacted at the molding dry unit weight and variable water content, as presented in Table 1. For all curing conditions, $q_{t}$ increases systematically as $\eta /C_{iv}^{0.50}$ decreases, reflecting the dominant influence of reduced porosity and higher volumetric cement content on bond formation and tensile resistance. At low water contents (9.8–10.2%), where the mixtures exhibit both lower porosities (39–45%) and reduced $\eta /C_{iv}^{0.50}$ values (19–31), $q_{t}$ reaches its highest levels, typically ranging between 100 kPa and 260 kPa, with peak values exceeding 250 kPa for $\eta /C_{iv}^{0.50}$ = 19.3. As moisture increases to intermediate levels (14–19%), $\eta /C_{iv}^{0.50}$ rises to the range of 22–46, and $q_{t}$ correspondingly decreases, producing tensile strengths mostly between 20 kPa and 120 kPa. At high molding water contents (24–34%), the index remains elevated (26–46) due to increased porosity and reduced dry density, and $q_{t}$ drops significantly, generally falling within 12–60 kPa, even with comparable cement contents. The results show a clear inverse and nonlinear relationship: lower $\eta /C_{iv}^{0.50}$ values (indicating denser structures and higher cement effectiveness) consistently yield higher $q_{t}$, whereas increases in moisture content promote higher porosity and weaker interparticle bonding, reducing tensile strength.

Table 7 describes the equations governing the unconfined compressive and splitting tensile strengths of compacted soil–cement blends.

### 4.3. Normalization Equations for Estimating the Unconfined Compressive and Splitting Tensile Strength

**Figure 6:**
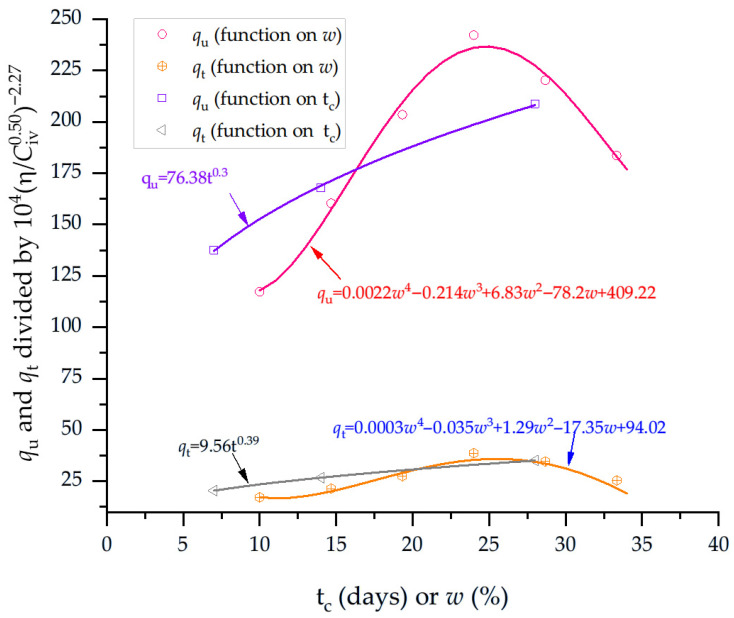
Normalization equations to estimate the splitting tensile strength and compressive strength of soil–cement blends, considering the curing times and water contents.

The normalization process begins by dividing the porosity–cement ratio by the corresponding water content values. These results are then further normalized with respect to the curing time (t_c_). In this way, a single equation is developed to estimate both the unconfined compressive strength and the splitting tensile strength results. Thus, Figure 6 presents the normalization of $q_{u}$ and $q_{t}$ results in a unique equation. The equation form is as follows:

(8) $$q_{u\;}and\;q_{t}=w\;function{\left[\frac{\eta }{{\left(C_{iv}\right)}^{x}}\right]}^{B}$$

where *w* is a function of the water content. For soil–cement blends compacted in different water content and dry unit weights, the general equations for estimating the strength of compacted blends are as follows for $q_{u}$ and $q_{t}$, respectively:

(9) $$q_{u\;}=\left[0.0022\omega^{4}-0.214\omega^{3}+6.83\omega^{2}-72.8\omega +409\right]\times 10^{4}{\left[\frac{\eta }{{\left(C_{iv}\right)}^{0.50}}\right]}^{-2.27}$$

(10) $$q_{t\;}=\left[0.0003\omega^{4}-0.035\omega^{3}+1.29\omega^{2}-17.35\omega +94.02\right]\times 10^{4}{\left[\frac{\eta }{{\left(C_{iv}\right)}^{0.50}}\right]}^{-2.27}$$

**Figure 7:**
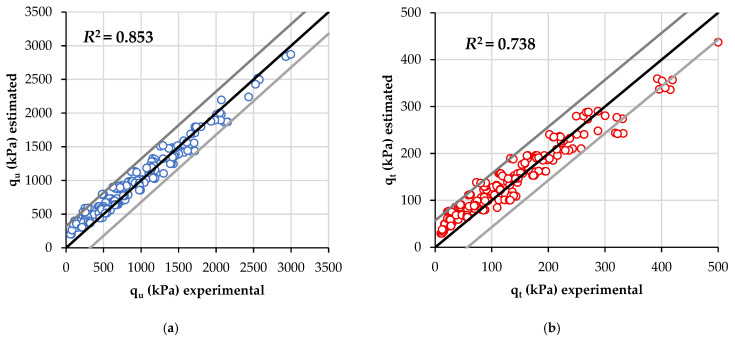
Results considering the normalized equations when mixes were compacted at conditions described in Table 1 of estimated: (**a**) compressive strength; and (**b**) splitting tensile strength.

For validating the equations, experimental values of water content, porosity, and C_iv_ for each specimen were replaced in Equations (9) and (10) with compressive and tensile strength (when corresponding). Thus, Figure 7 provides the *R*^2^ adjusted values of the estimating equations. Both $q_{u}$ and $q_{t}$ general equations yielded high values of *R*^2^ (above 0.700), specifically *R*^2^ = 0.853 (compressive) and *R*^2^ = 0.738 (splitting tensile).

For soil–cement blends compacted in OMC and MDD, the general equations for estimating the strength of compacted blends are as follows for $q_{u}$ and $q_{t}$, respectively:

(11) $$q_{u\;}=76.38t_{c}^{0.30}\times 10^{4}{\left[\frac{\eta }{{\left(C_{iv}\right)}^{0.50}}\right]}^{-2.27}$$

(12) $$q_{t\;}=9.56t_{c}^{0.39}\times 10^{4}{\left[\frac{\eta }{{\left(C_{iv}\right)}^{0.50}}\right]}^{-2.27}$$

**Figure 8:**
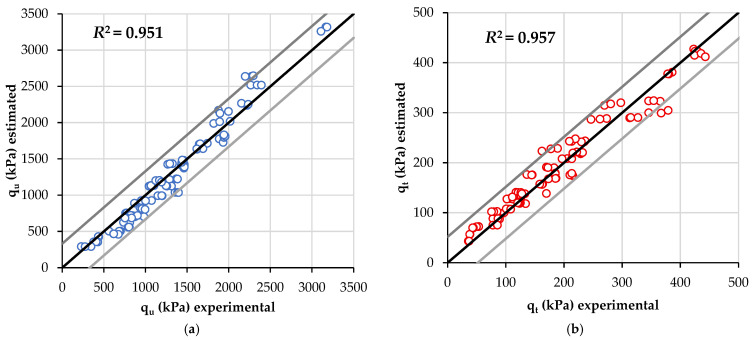
Results considering the normalized equations when mixes were compacted at OMC and MDD of estimated: (**a**) compressive strength; and (**b**) splitting tensile strength.

Equations (11) and (12) are a function of curing time. To validate the equations, experimental values for curing time, porosity, and C_iv_ for each specimen were substituted into Equations (11) and (12) for compressive and tensile strength, respectively. Thus, Figure 8 provides the *R*^2^ adjusted values for the estimating equations. Both $q_{u}$ and $q_{t}$ general equations yielded high values of *R*^2^, specifically *R*^2^ = 0.951 (compressive) and *R*^2^ = 0.957 (splitting tensile).

### 4.4. Machine Learning Results

The eight algorithm categories were evaluated together with their internal variants, resulting in a total of twenty-eight machine learning presets. The root-mean-square error (RMSE) and the coefficient of determination ($R^{2}$) were computed for each preset during both the validation and testing stages for the two response variables, $q_{u}$ and $q_{t}$, as shown in Table 8. The cells highlighted in gray correspond to the best-performing model for each response. Overall, the results indicate that the Matern 5/2 Gaussian Process Regression (GPR) model provides the most accurate predictions for both responses, yielding the lowest RMSE values and $R^{2}$ values approaching unity. Accordingly, RMSE values of 61.8 and 10.5 were obtained for $q_{u}$ during the validation and testing stages, respectively, whereas RMSE values of 12.2 and 12.6 were obtained for $q_{t}$ in these stages. Similarly, the $R^{2}$ values were close to 1. During the validation stage, a value of 0.963 was obtained for $q_{u}$, while the testing stage reached a value of 0.997. For $q_{t}$, the corresponding $R^{2}$ values were 0.984 and 0.988 for the validation and testing stages, respectively. The robust linear method was the only approach to yield negative *R*^2^ values during the training phase for $q_{u}$. No negative values were observed for $q_{t}$ at this stage.

In general, the different GPR variants exhibited excellent performance, demonstrating high predictive capability and robust generalization. It is also worth noting that several neural network architectures, particularly the wide and multilayer configurations, achieved competitive accuracy, with RMSE and $R^{2}$ values comparable to those of the GPR models. Linear models presented poor generalization to unseen data. Tree-based models exhibited moderate performance; fine and boosted trees performed reasonably well, whereas medium and coarse trees showed reduced accuracy. Support Vector Machines offered mixed results for $q_{u}$ and $q_{t}$.

For the selected GPR model, the selected hyperparameters are summarized as follows: constant basis function; Matern 5/2 kernel function; use of an isotropic kernel with automatic length-scale determination; automatic estimation of the noise variance (sigma) and signal standard deviation; and activation of data standardization and numerical hyperparameter optimization options.

**Figure 9:**
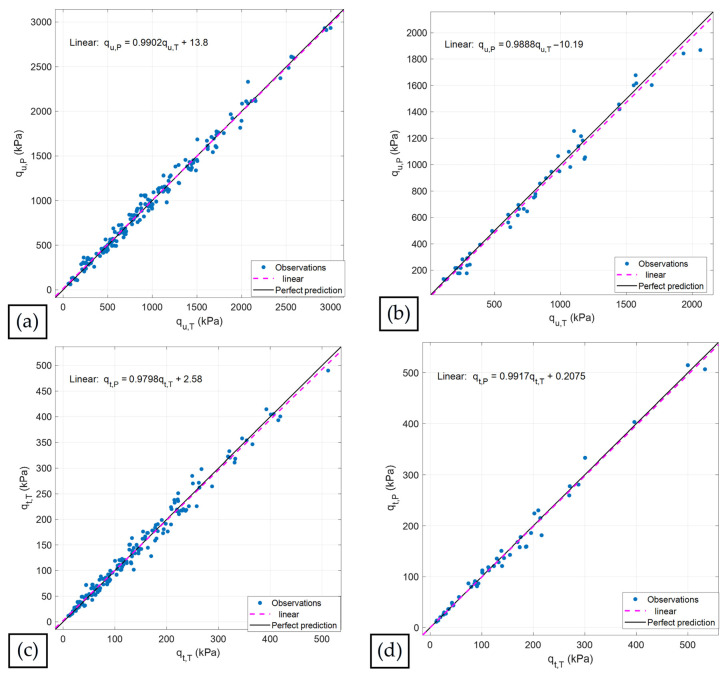
Comparison between true and predicted values for (**a**) $q_{u}$ during the validation stage; (**b**) $q_{u}$ during the testing stage; (**c**) $q_{t}$ during the validation stage; and (**d**) $q_{t}$ during the testing stage.

Figure 9 illustrates the predictive capability of the GPR model for $q_{u}$ and $q_{t}$. The trained dataset consists of 240 samples collected during the experimental program, of which 80% were used for validation and the remaining samples for testing. Figure 9a,b compares the true values (experimental samples) with the predicted values obtained using the Matern 5/2 GPR model for $q_{u}$. The black line represents the perfect-fit condition, whereas the magenta dashed line shows the linear trend of the dataset. Both lines exhibit nearly identical behavior, demonstrating an excellent level of agreement. For the validation stage, these results correspond to the following expression:

(13) $$q_{u,P\;}=0.9902q_{u,T}+13.8$$

where T corresponds to the true values, and P represents the values predicted by the Matern 5/2 GPR model.

A similar interpretation applies to the results for $q_{t}$, as the Matern 5/2 GPR preset also provides highly accurate predictions for this response. Once again, the black and magenta dashed lines are practically parallel, reflecting the excellent agreement between the true and predicted values during both the validation and testing stages, as shown in Figure 9c,d.

**Figure 10:**
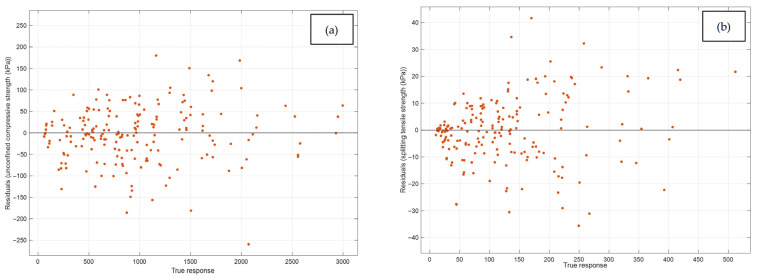
Comparison between residuals and true responses for the Matern 5/2 GPR model for the validation stage: (**a**) $q_{u}$ and (**b**) $q_{t}$.

Figure 10 shows that the residuals are predominantly concentrated around zero over the entire range of true response values, indicating essentially unbiased predictive behavior of the model. Most of the residuals fall within the interval −100 to 100 kPa, confirming that the prediction errors remain limited for the vast majority of the samples and that no systematic overestimation or underestimation is evident across the response domain.

**Figure 11:**
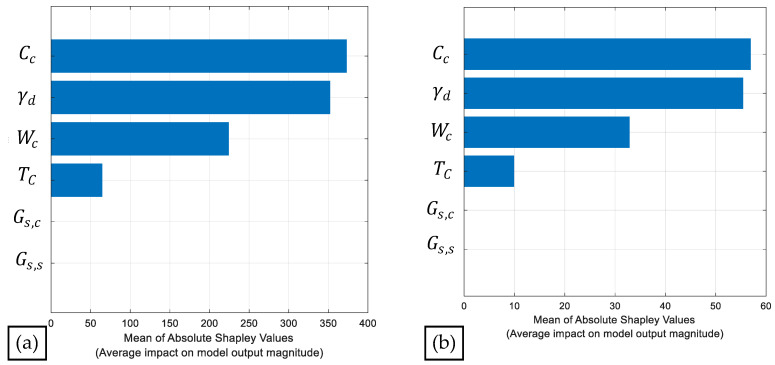
Computation of Shapley values for (**a**) $q_{u}$ and (**b**) $q_{t}$.

In addition, Shapley values were computed to identify the most influential predictors for estimating $q_{u}$ and $q_{t}$, as shown in Figure 11. Based on the average impact on the model output (mean absolute Shapley values), the most relevant predictors, in descending order, are $C_{c}$, $\gamma_{d}$, $W_{c}$, and $T_{c}$ for both responses. The remaining predictors ($G_{s,c}$ and $G_{s,s}$) exhibited very low influence, indicating a weak dependency of the model on these variables.

**Figure 12:**
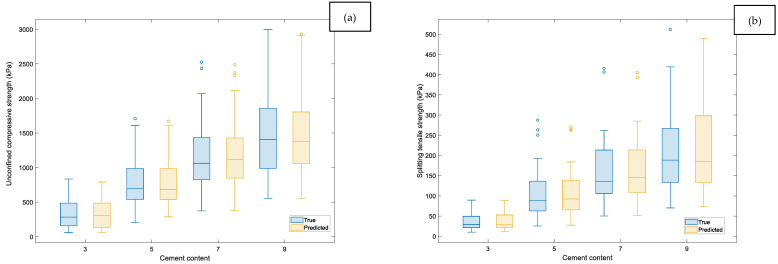
Boxplot analysis for cement content: (**a**) $q_{u}$ and (**b**) $q_{t}$.

Considering that the cement content is the most sensitive predictor according to the Shapley values, the boxplots analyzed in Figure 12 show a strong level of consistency between the true and predicted responses. For both $q_{u}$ and $q_{t}$, the medians of the predicted values closely follow those of the experimental data across the full cement-content range (approximately 300–1600 kPa for $q_{u}$ and 20–180 kPa for $q_{t}$). The interquartile ranges also exhibit comparable widths, indicating that the model adequately captures the variability of the responses. The substantial overlap between the true and predicted distributions confirms the robustness and reliability of the Matern 5/2 GPR model predictions across the full parameter range.

A comparison was made between the porosity-to-cement model and the Matern 5/2 GPR model to examine the order of magnitude of their predictions, as shown in Table 9. The comparative analysis between the porosity–cement index and the Matern 5/2 Gaussian Process Regression (GPR) model demonstrates the clear superiority of ML-based approaches for predicting the mechanical behavior of cemented silt. While the porosity–cement formulations yielded moderate to strong correlations (*R*^2^ = 0.738–0.957), the Matern 5/2 GPR achieved substantially higher accuracy, reaching *R*^2^ values up to 0.997 for q_n_ and 0.988 for q_t_ in testing. This improvement reflects the model’s ability to capture nonlinear interactions between porosity, cement content, molding water content, and curing processes—relationships that the traditional index cannot fully represent. The robustness of the GPR model across validation and testing scenarios (*R*^2^ > 0.96) confirms its high generalization potential, offering reliable predictions even under varying compaction and chemical conditions. These findings highlight the growing importance of machine learning in soil stabilization, where ML can complement classical indices by providing more precise, data-driven insight into strength evolution, enabling optimized mix design and reducing experimental cost and uncertainty in geotechnical engineering.

It of utmost importance to highlight that ML models must be used considering the range of applications where these models were trained, validated, and tested, which corresponds to the information presented in Table 1 and Table 2. Considering the dataset used in this study, the cross-fold validation method was employed to mitigate the risk of overfitting. In addition, not only did the validation stage yield satisfactory results but the testing stage also confirmed that the selected machine learning model is robust and not affected by overfitting (see the Matern 5/2 GPR ML method in Table 8).

## 5. Conclusions

- The porosity–cement index (η/C_iv_) proved to be a robust and unifying parameter for predicting the mechanical behavior of the cemented silt, exhibiting strong correlations for both $q_{u}$ and $q_{t}$. For 28-day curing, the best-fit exponent converged to *x* = 0.50, producing high determination coefficients (*R*^2^ = 0.98 for $q_{u}$ and *R*^2^ = 0.97 for $q_{t}$), confirming the validity of a power-law relationship for materials compacted under Standard, Intermediate, and Modified energies.
- Mechanical strength increased markedly with decreasing η/C_iv_, demonstrating the dominant influence of porosity reduction over cement volume fraction. For $q_{u}$, mixtures with η/C_iv_ = 45–46 exhibited very low strengths (60–80 kPa), whereas reducing η/C_iv_ to 19–21 yielded $q_{u}$ between 1500 and 3000 kPa, representing 15–25-fold increases. For $q_{t}$, the same trend was observed: at η/C_iv_ = 46, $q_{t}$ remained below 15 kPa, while values of 22–23 produced $q_{t}$ between 130 and 330 kPa, and η/C_iv_ = 19 yielded peak strengths above 400 kPa, confirming a consistent strengthening mechanism for both tensile and compressive responses.
- Compaction water content played a critical role in defining the porosity–cement state and the corresponding strength envelope. Specimens molded at *w* = 10.0–10.2% achieved the lowest porosities (39%) and the highest $q_{u}$ values, reaching 1500–3000 kPa depending on *C*_iv_. In contrast, increasing the water content to 19–24% raised the porosity to 50–51%, resulting in a strength below 120 kPa for the duplicate cement content. This demonstrates the strong coupling between molding water content, packing structure, and cementation efficiency.
- Machine learning models (Gaussian Process Regression, Matern 5/2 kernel) outperformed the empirical porosity–cement model in prediction accuracy, achieving *R*^2^ = 0.963 (validation) and *R*^2^ = 0.997 (testing) for $q_{u}$ and *R*^2^ = 0.984–0.988 for $q_{t}$. The ML models captured nonlinear interactions among moisture, density, curing age, and binder content that are not explicitly represented in the η/C_iv_ formulation. However, when used together, both approaches provide complementary insights: η/C_iv_ explains the mechanics, while ML enhances predictive precision.
- The combined framework of porosity–cement index + machine learning offers a robust dual methodology for the design of cemented silt geomaterials, enabling both mechanistic understanding and high-accuracy prediction. This study demonstrates that η/C_iv_ efficiently generalizes physical behavior across compaction energies and moisture states, while ML provides superior prediction for engineering applications. This integrated approach significantly reduces experimental effort and enables the optimization of mix designs for sustainable ground improvement.
- While ML algorithms provide superior predictive accuracy, η/C_iv_ offers a mechanistic explanation of strength development across varying compaction states. The combined framework demonstrates that physically based indices and data-driven models are complementary rather than redundant, providing a practical and robust methodology for the design and optimization of cement-stabilized soils.

## Figures and Tables

**Table 1:** Mixed proportion design for soil–cement compacted blends cured under 28 days at non-optimal compaction conditions (i.e., varying *γ*_d_ and *w*).

Molding *γ*_d_ (kN/m^3^)	Soil (%)	C (%)	Molding *w* (%)	*t*_c_ (Days)	Specimens	Test
13.0	100	3	10, 14.67, 19.33, 24, 28.67, and 33.34	28	36	$q_{u}$ $\;\mathrm{and}\;q_{t}$
	100	5	10, 14.67, 19.33, 24, 28.67, and 33.34	28	36	$q_{u}$ $\;\mathrm{and}\;q_{t}$
	100	7	10, 14.67, 19.33, 24, 28.67, and 33.34	28	36	$q_{u}$ $\;\mathrm{and}\;q_{t}$
	100	9	10, 14.67, 19.33, 24, 28.67, and 33.34	28	36	$q_{u}$ $\;\mathrm{and}\;q_{t}$
13.75	100	3	28.67 and 33.34	28	12	$q_{u}$ $\;\mathrm{and}\;q_{t}$
	100	5	28.67 and 33.34	28	12	$q_{u}$ $\;\mathrm{and}\;q_{t}$
	100	7	28.67 and 33.34	28	12	$q_{u}$ $\;\mathrm{and}\;q_{t}$
	100	9	28.67 and 33.34	28	12	$q_{u}$ $\;\mathrm{and}\;q_{t}$
14.5	100	3	10, 14.67, 19.33, 24, and 28.67	28	30	$q_{u}$ $\;\mathrm{and}\;q_{t}$
	100	5	10, 14.67, 19.33, 24, and 28.67	28	30	$q_{u}$ $\;\mathrm{and}\;q_{t}$
	100	7	10, 14.67, 19.33, 24, and 28.67	28	30	$q_{u}$ $\;\mathrm{and}\;q_{t}$
	100	9	10, 14.67, 19.33, 24, and 28.67	28	30	$q_{u}$ $\;\mathrm{and}\;q_{t}$
16.0	100	3	10, 14.67, 19.33, and 24	28	24	$q_{u}$ $\;\mathrm{and}\;q_{t}$
	100	5	10, 14.67, 19.33, and 24	28	24	$q_{u}$ $\;\mathrm{and}\;q_{t}$
	100	7	10, 14.67, 19.33, and 24	28	24	$q_{u}$ $\;\mathrm{and}\;q_{t}$
	100	9	10, 14.67, 19.33, and 24	28	24	$q_{u}$ $\;\mathrm{and}\;q_{t}$

**Table 2:** Mixed proportion design for soil–cement blends cured under 7, 14, and 28 days at optimal compaction conditions (i.e., OWC and MDD).

Energy	Soil (%)	C (%)	*OWC* (%)	*MDD* (kN/m^3^)	*t*_c_ (Days)	Specimens	Test
Standard	100	3	26	13.85	7, 14, and 28	18	$q_{u}$ $\;\mathrm{and}\;q_{t}$
	100	5	26.5	13.80	7, 14, and 28	18	$q_{u}$ $\;\mathrm{and}\;q_{t}$
	100	7	26	14	7, 14, and 28	18	$q_{u}$ $\;\mathrm{and}\;q_{t}$
	100	9	25.5	14	7, 14, and 28	18	$q_{u}$ $\;\mathrm{and}\;q_{t}$
Intermediate	100	3	18	15.65	7, 14, and 28	18	$q_{u}$ $\;\mathrm{and}\;q_{t}$
	100	5	18	15.55	7, 14, and 28	18	$q_{u}$ $\;\mathrm{and}\;q_{t}$
	100	7	18.5	15.55	7, 14, and 28	18	$q_{u}$ $\;\mathrm{and}\;q_{t}$
	100	9	18	15.55	7, 14, and 28	18	$q_{u}$ $\;\mathrm{and}\;q_{t}$
Modified	100	3	15	16.85	7, 14, and 28	18	$q_{u}$ $\;\mathrm{and}\;q_{t}$
	100	5	15	17.05	7, 14, and 28	18	$q_{u}$ $\;\mathrm{and}\;q_{t}$
	100	7	14.5	16.95	7, 14, and 28	18	$q_{u}$ $\;\mathrm{and}\;q_{t}$
	100	9	15	16.95	7, 14, and 28	18	$q_{u}$ $\;\mathrm{and}\;q_{t}$

**Table 3:** Interpretability of categories of machine learning models.

Model Type (Presets)	Interpretability	
	Easy	Hard
Linear (Linear, Interactions, Robust, and Stepwise)	∎	
Decision Trees (Fine, Medium, and Coarse)	∎	
Support Vector Machine—SVM (Linear, Quadratic, Cubic, Fine Gaussian, Medium Coarse, and Coarse Gaussian)	∎ (linear SVM)	∎ (other kernels)
Efficiently Trained Linear (Least Squares and Linear SVM)	∎	
Gaussian Process Regression (Squared Exponential, Matern 5/2, Exponential, and Rational Quadratic)		∎
Kernel models (SVM and Least Squares)		∎
Ensembles of Trees (Boosted and Bagged)		∎
Neural Networks (Narrow, Medium, Wide, Bilayered, and Trilayered)		∎

**Table 4:** Formulas of machine learning models.

Model Type	Formula	Equation No.	Observation	Notation
Linear	$y=\beta_{0}+\beta_{1}x+\varepsilon$	(2)		y = response, x = predictor, $\beta_{0}$ = intersection term, $\beta_{1}$ = slope term, and ε = error term.
Trees	Not presented	-	Prediction begins at the root node and proceeds down to a leaf node.	None.
Support Vector Machine (SVM)	$f\left(x\right)=\sum_{n=1}^{N}\left(\alpha_{n}-\alpha_{n}^{*})G(x_{n},x\right)+b$	(3)	It is used for nonlinear SVM regression.	$G(x_{n},x)$ = kernel function, $f\left(x\right)$ = function for computing new values, N = observations, and b = bias term.
Efficiently Trained Linear	Not presented	-	Corresponds to linear least squares models and linear SVM.	None.
Gaussian Process Regression	$P(y|f,\;X)\sim N(y|H\beta +f,\sigma^{2}I)$	(4)	None.	β = coefficient that depends on data, y = response, x = predictors, N = Gaussian distribution, $\sigma^{2}$ = variance, and H = vector of basis functions.
Kernel Models	$f\left(x\right)=\sum_{i=1}^{N}\alpha_{i}K(x_{i},x)$	(5)	None.	$f\left(x\right)$ = function for computing new values, $K(x_{i},x)$ = kernel function, and $\alpha_{i}$ = coefficients.
Ensembles of Trees	$f\left(x\right)=\sum_{t=1}^{T}\alpha_{t}h_{t}(x)$	(6)	Applicable for the boosting algorithm.	$f\left(x\right)$ = function for computing new values, $\alpha_{t}$ = weights of the weak hypothesis, and $h_{t}(x$) = prediction of learner with index t.
Neural Networks	$a^{3}=f^{3}({LW}^{3,2}f^{2}\left({LW}^{2,1}f^{1}\left({IW}^{1,1}p+b^{1}\right)+b^{2}\right)+b^{3})$	(7)	It corresponds to the trilayered neural network.	a = output vector, p = input vector, W = refers to weight, I = input matrix, and L = layer matrix. The numbers refer to the layers.

**Table 5:** Identification of predictors and responses for applying ML models.

Variables	Identification	Source
Predictors (inputs obtained from experimental program)	$W_{c}$ = water content. $\gamma_{d}$ = unit dry weight. $C_{c}$ = cement content. $G_{s,c}$ = specific gravity content. $G_{s,s}$ = specific gravity of soil. $T_{c}$ = curing time.	Experimental Program (Section 2).
Responses (outputs computed by ML models)	$q_{u}$ = unconfined compressive strength. $q_{t}$ = splitting tensile strength.	Machine learning models (Section 3).

**Table 6:** Results of equations for estimating unconfined compressive and splitting tensile strength of soil–cement compacted blends, derivation of porosity-to-cement ratio index, and considering the OMC and OWC (Figure 3).

Test	Curing Time	A Value (×10^4^)	*x* Exponent	*B* Exponent	*R* ^2^
$q_{u}$	7	137.61	0.50	−2.27	0.915
	14	167.86	0.50	−2.27	0.891
	28	208.83	0.50	−2.27	0.956
$q_{t}$	7	20.45	0.50	−2.27	0.944
	14	26.75	0.50	−2.27	0.953
	28	35.14	0.50	−2.27	0.959

**Table 7:** Results of equations for estimating unconfined compressive and splitting tensile strength of soil–cement compacted blends, derivation of porosity-to-cement ratio index, and considering the variation in water content and dry unit weight (Figure 4 and Figure 5).

Test	Water Content	A Value (×10^4^)	*x* Exponent	*B* Exponent	*R* ^2^
$q_{u}$	10.00	117.22	0.50	−2.27	0.920
	14.67	160.23	0.50	−2.27	0.914
	19.33	203.29	0.50	−2.27	0.942
	24.00	241.92	0.50	−2.27	0.975
	28.67	220.01	0.50	−2.27	0.971
	33.34	183.32	0.50	−2.27	0.965
$q_{t}$	10.00	17.19	0.50	−2.27	0.905
	14.67	21.44	0.50	−2.27	0.863
	19.33	27.51	0.50	−2.27	0.893
	24.00	38.72	0.50	−2.27	0.920
	28.67	34.63	0.50	−2.27	0.933
	33.34	25.41	0.50	−2.27	0.914

**Table 8:** Comparison of statistical performance measures for the machine learning models.

Preset	$q_{u}$				$q_{t}$			
	RMSE (V)	*R*^2^ (V)	RMSE (T)	*R*^2^ (T)	RMSE (V)	*R*^2^ (V)	RMSE (T)	*R*^2^ (T)
Linear	305.1	0.103	242.2	−0.462	43.2	0.803	56.2	0.765
Interactions Linear	258.3	0.357	223.3	−0.242	26.1	0.928	33.9	0.914
Robust Linear	326.4	−0.026	201.0	−0.007	46.6	0.772	63.6	0.698
Stepwise Linear	256.2	0.368	226.6	−0.279	26.7	0.925	33.7	0.915
Fine Tree	154.1	0.771	60.5	0.909	41.7	0.817	38.7	0.889
Medium Tree	221.5	0.527	138.6	0.522	51.6	0.719	57.1	0.757
Coarse Tree	295.3	0.160	200.6	−0.002	68.0	0.513	82.0	0.499
Linear SVM	321.9	0.002	206.8	−0.066	45.1	0.786	60.8	0.725
Quadratic SVM	223.3	0.520	190.4	0.096	23.7	0.941	30.7	0.930
Cubic SVM	213.0	0.563	118.3	0.651	98.3	−0.016	61.3	0.721
Fine Gaussian SVM	265.5	0.321	145.2	0.475	33.9	0.879	46.5	0.839
Medium Gaussian SVM	277.7	0.257	175.3	0.234	23.7	0.941	26.5	0.948
Coarse Gaussian SVM	320.5	0.010	205.8	−0.055	51.0	0.726	66.7	0.669
Efficient Linear Least Squares	304.9	0.104	240.3	−0.439	48.1	0.756	61.1	0.722
Efficient Linear SVM	320.3	0.012	205.3	−0.050	73.5	0.431	88.7	0.414
Boosted Trees	109.9	0.884	51.0	0.935	26.8	0.925	33.9	0.914
Bagged Trees	177.6	0.696	105.6	0.722	38.6	0.843	48.0	0.828
Squared Exponential GPR	58.0	0.968	11.2	0.997	13.9	0.980	12.8	0.988
Matern 5/2 GPR	61.8	0.963	10.5	0.997	12.2	0.984	12.6	0.988
Exponential GPR	55.7	0.970	12.8	0.996	13.9	0.980	10.9	0.991
Rational Quadratic GPR	61.7	0.963	10.3	0.997	12.3	0.984	12.4	0.989
Narrow NN	154.3	0.771	118.4	0.651	21.9	0.949	25.3	0.952
Medium NN	60.1	0.965	25.2	0.984	15.3	0.975	14.6	0.984
Wide NN	35.4	0.988	15.0	0.994	14.6	0.978	11.9	0.990
Bilayered NN	106.0	0.892	97.2	0.765	19.0	0.962	13.8	0.986
Trilayered NN	73.0	0.949	25.4	0.984	17.2	0.969	14.4	0.984
SVM Kernel	321.6	0.004	204.6	−0.043	92.4	0.101	120.2	−0.076
Least Squares Regression Kernel	166.2	0.734	82.9	0.829	32.8	0.887	63.6	0.699

Note: V = validation, T = Test, NN = neural network, and GPR = Gaussian Process Regression.

**Table 9:** Comparison of *R*^2^ values between porosity-to-cement and Matern 5/2 models.

Response	Porosity-to-Cement Model		Matern 5/2 GPR Model	
	Equation	*R* ^2^	*R* ^2^	
			Validation	Testing
$q_{u}$	Equation (9)	0.951	0.963	0.997
$q_{t}$	Equation (10)	0.957	0.984	0.988
$q_{u}$	Equation (11)	0.853	0.963	0.997
$q_{t}$	Equation (12)	0.738	0.984	0.988

## Data Availability

The original contributions presented in this study are included in the article/Supplementary Material. Further inquiries can be directed to the corresponding authors.

## Supplementary Materials

The following supporting information can be downloaded at: https://www.mdpi.com/article/10.3390/ma19030498/s1.
